# Factors Associated with the Transition Time to Full Enteral Feeding in Newborns with Hypoxic Ischemic Encephalopathy

**DOI:** 10.34172/aim.2022.87

**Published:** 2022-08-01

**Authors:** Asli Okbay Gunes, Nilgun Karadag, Sevilay Topcuoglu, Elif Ozalkaya, Handan Hakyemez Toptan, Emre Dincer, Hakan Cakir, Guner Karatekin

**Affiliations:** ^1^University of Health Sciences, Zeynep Kamil Maternity and Children’s Training and Research Hospital -Istanbul, Turkey

**Keywords:** Enteral feeding, Hypothermia, Hypoxic-ischemic encephalopathy, Neonates

## Abstract

**Background:**

We aimed to assess the factors associated with the transition time to full enteral feeding (FEF) in newborns with hypoxic ischemic encephalopathy (HIE) undergoing therapeutic hypothermia.

**Methods:**

We obtained data retrospectively from medical records of the neonates diagnosed with HIE and treated by therapeutic hypothermia to evaluate the factors associated with transition time to FEF.

**Results:**

Sixty-one neonates were included in the study. The median gestational age (GA) and birth weight were 39 (37–40) weeks and 3245 (2715–3575) grams, respectively. APGAR scores at the first and fifth minutes were 3 (1–5) and 6 (4–7), respectively. Fifty-seven (93.4%) of the newborns were diagnosed as having moderate HIE, and 4 (6.6%) of them had severe HIE. Transition time to FEF was found to be negatively correlated with gestational week (*r*, *P*: -0.280, 0.029) and birth weight (*r*, *P*: -0.315, 0.013); and positively correlated with lactate (*r*, *P*: 0.295, 0.044), BUN (*r*, *P*: 0.285, 0.026) and creatinine levels (*r, P*: 0.345, 0.007); duration of invasive (*r*, *P*: 0.565, 0.0001) and non-invasive mechanical ventilation (*r*, *P*: 0.261, 0.042), use of antibiotics (*r*, *P*: 0.556, 0.0001) and inotropic agents (*r*, *P*: 0.524, 0.0001) and hospitalization (*r*, *P*: 0.654, 0.0001).

**Conclusion:**

Clinicians should be more careful while starting to feed babies undergoing therapeutic hypothermia with higher lactate levels and impaired renal functions, and should be encouraged to feed clinically stable neonates with HIE as soon as possible, as the transition time to FEF could be related with better clinical outcomes.

## Introduction

 Hypoxic-ischemic encephalopathy (HIE) is an important cause of morbidity and mortality in newborns. The incidence of HIE is 1 to 8 per 1000 live births in developed countries and as high as 26/1000 live births in underdeveloped countries.^1^ Among 19 857 live births, 93 babies were diagnosed as having HIE in Turkey as reported by the Turkish Neonatal Society Hypoxic Ischemic Encephalopathy Study Group; the incidence was found to be 2.6 per 1000 live births and 1.2% in patients hospitalized in intensive care units.^2^

 Therapeutic hypothermia (TH) is the only proven treatment option and causes a reduction in the rates of mortality and neurologic sequelae in neonates who are diagnosed as having moderate and severe HIE.^3^ TH is also thought to have favorable effects on gastrointestinal morbidity and feeding tolerance, but there is no agreement on the type, volume or frequency of enteral feeds given to these patients and this results in variations in enteral feeding strategies for these infants.^4-6^ There is scanty evidence indicating that hypothermia and enteral feeding may have a protective effect on the gastrointestinal system after hypoxic insult, and careful introduction of enteral feeds seems to be well-tolerated and safe.^7^ A cohort study including HIE patients, who received delayed enteral feeding in the United Kingdom and early enteral feeding during TH in Sweden, showed that enteral feeding during TH was not correlated with increased mortality or morbidity.^8^ These data show that early minimal enteral nutrition (MEN) during TH is reasonable and does not lead to significant complications. It was also determined that MEN during TH was related to a shortened length of hospital stay and shortened time to full enteral feeding (FEF), and did not cause a rise in feeding complications or systemic inflammation.^9^

 We hypothesized that enteral feeding success could be associated with some of the clinical and laboratory findings and clinical outcomes of neonates undergoing TH. We aimed to evaluate the factors associated with transition time to FEF in neonates undergoing TH.

## Materials and Methods

 We obtained data retrospectively from medical records of the neonates diagnosed with moderate and severe HIE, and treated by TH between January 2015 and June 2020 to evaluate the factors associated with the transition time to FEF. Hypothermia treatment decision was made according to the Turkish Neonatal Society Guideline on neonatal encephalopathy recommendations.^10,11^ Treatment criteria were as follows: (1) newborns with a gestational age (GA) of ≥ 36 weeks and aged below ≤ 6 hours; a pH value of ≤ 7.00 or BE value of ≤ -16 mmol/L in the blood sample collected from the cord or in the blood sample collected from the baby in the first hour after delivery; (3) a tenth minute APGAR score of < 5 or persisting need for resuscitation; (4) signs of moderate or severe encephalopathy on clinical evaluation. Cooling to a rectal temperature of 33.5 ± 0.5°C was achieved in all infants. Rewarming to 36.5°C was started following 72-hour cooling by elevating the temperature with a rate of 0.5°C per hour. The Sarnat and Sarnat staging system was used to determine the stage of HIE.^10,11^ GA was estimated based on the postmenstrual date (PMD) or if PMD was not known, Ballard score was used to estimate GA.^12^ Rectal temperature, blood pressure, oxygen saturation, heart rate, respiratory rate, fluid balance and amplitude integrated electroencephalogram monitoring were recorded consistently. The respective clinical team determined clinical management. The neonates who died before tolerating FEF and who could not tolerate enteral feeding because of any medical condition other than HIE, were excluded from the study.

 Data about the antenatal and perinatal history, APGAR scores, sex, birth weight, all medical treatments, respiratory support modes, ultrasonography and echocardiography findings were extracted from the hospital records in a retrospective manner. All laboratory results including blood gases, complete blood count, prothrombin time (PT), international normalized ratio (INR), activated partial thromboplastin time (aPTT) on admission to intensive care unit; aspartate aminotransferase (AST), alanine aminotransferase (ALT), blood urea nitrogen (BUN), creatinine, uric asid, creatinine kinase (CK), creatinine kinase-muscle brain (CK-MB), lactate dehydrogenase (LDH) and troponin I in the 24^th^ hour of life were recorded. Extensive data related to fluids and nutrition were recorded for the first four days, because this period enclosed the duration of TH and rewarming. Complications such as necrotising enterocolitis (NEC) and sepsis, and the incidence of persistent pulmonary hypertension were recorded. The transition time to FEF was specified as the time between birth and the time when application of intravenous fluid was stopped. Length of hospital stay was specified as the time between admission to the unit and discharge from the unit. There were some missing data that we could not obtain from the patients’ records. The APGAR scores of three neonates were not found in their files. PT, INR, aPTT ​​of 12 patients were not measured on admission; CK and CK-MB values ​​of four patients, LDH value of one patient, and troponin I values of 17 patients were not measured at the postnatal 24th hour.

 Fluid balance was maintained with 10% dextrose infusion in at least 72 hours of life. Minimal enteral feeding was started as soon as the neonates were considered stable. Neonates with normal blood pressure (even with inotropic support) and blood gas parameters (even with ventilator support), and urine output above 1 mL/kg/h were considered clinically stable. Breast milk was withheld for the first 24 hours of life, and if it was not available, artificial formula was started after the first 24 hours. Donor milk was unavailable due to lack of donor milk banks in our country. During TH, babies were fed with an orogastric tube and routine residual control was performed. Minimal enteral feedings were initiated as < 20 mL/kg/d. Enteral feeding was terminated, if abdominal distention and large ( > 5 mL/kg), bilious or blood stained gastric residuals were observed. The volume of enteral feeding was increased, if the infant tolerated the feeds. Enteral feeding was increased at a rate of 10–20 mL/kg/d as tolerated. Enteral feeding was described as milk feeds of any type (expressed maternal breast milk or artificial formula) administered by any route (orogastric tube, bottle and suckling at the breast). If enteral feeding could not be started in three days, parenteral nutrition was started. As this was an observational study, it would be difficult to find out the risk factors leading to longer transition time to FEF and the factors affected by the time to FEF. Therefore, only the temporal relationships between time to FEF and factors that might be associated with FEF were planned to be assessed. Abnormal laboratory results reflecting possible hypoxic ischemic organ damage and worse clinical course reflecting more severe HIE were accepted as risk factors for longer time to FEF. The duration of hospital stay was accepted as a factor affected by the time to FEF, but it might actually be affected by the severity of HIE.

###  Statistical Analysis

 Statistical analyses were performed using the Statistical Package for Social Sciences version 21.0 (SPSS Inc., Chicago, IL, USA). Visual (histogram and probability graphics) and analytic methods (Kolmogorov-Smirnov/Shapiro-Wilk tests) were used to test the normality of the distribution of variables. The quantitative clinical characteristics of the newborns were expressed as mean and standard deviation for normally disturbed values or as median and interquartile range (IQR) for non-normally distributed variables. Categorical variables were expressed as frequencies. Non-parametric tests were used in the analyses since our data were not normally distributed. The Mann-Whitney U test was used to compare continuous variables by groups and Spearman’s correlation test was used to assess the correlations between variables. A *P* value < 0.05 was considered statistically significant.

## Results

 During the study period, a total of 65 neonates received TH for HIE. Four of the 65 neonates were excluded as three of them were diagnosed as having severe HIE and died before transition to FEF and one neonate with moderate HIE was diagnosed as having intestinal malrotation that did not allow him to tolerate enteral feeding before surgery. Fifty-seven (93.4%) of the newborns were diagnosed as having moderate HIE and four (6.6%) of them had severe HIE. The transition time to FEF was 7 (5–11) days for the whole group, 7 (5–10.5) days for the moderate HIE group and 19.5 (7.75–26.75) days for the severe HIE group (*P*= 0.08, Mann-Whitney U test). The length of hospital stay was 17 (12–31.5) days for the whole group, 16 (11.5–26) days for the moderate HIE group and 65 (49.25–77) days for the severe HIE group (*P* = 0.001, Mann-Whitney U test). No cases were diagnosed with NEC and culture-proven sepsis was not detected in any of the patients before tolerating FEF. Pulmonary hypertension was detected in three cases with moderate HIE and was successfully treated with medical therapy. The demographic, clinical and laboratory findings of the babies are shown in Table 1 and etiologies of HIE are shown in Table 2.

**Table 1:** Demographic, Clinical and Laboratory Findings of the Neonates with Hypoxic-ischemic Encephalopathy (*n* = 61)

**Demographic, Clinical and Laboratory Findings**	
Mother age, y (IQR)	28 (25–32)
Primiparous, n (%)	25 (41)
Vaginal delivery, n (%)	31 (50.8)
GA, week (IQR)	39 (37–40)
Birth weight, g (IQR)	3245 (2715–3575)
Female babies, n (%)	28 (45.9)
APGAR, first min (IQR)	3 (1–5)
APGAR, fifth min (IQR)	6 (4–7)
Moderate HIE/ Severe HIE, n (%)	57 (93.4)/ 4 (6.6)
pH, mm Hg (IQR)	6.98 (6.87–7.06)
Base excess, mmol/L (IQR)	-19 ([-22.85] – [-16.35])
Respiratory distress, n (%)	40 (47.5)
Convulsion, n (%)	29 (47.5)
Inotrope need, n (%)	27 (44.3)
Antibiotic use, days (IQR)	10 (7–14)
Transition time to full enteral feeding, day (IQR)	7 (5–11)
Length of hospital stay, days (IQR)	17 (12–31.5)

HIE, Hypoxic-ischemic encephalopathy; IQR, Interquartile range.

**Table 2:** Etiologies of Hypoxic-ischemic Encephalopathy in Newborns (*n* = 61)

	**n (%)**
Difficult birth	29 (47.5)
Meconium aspiration syndrome	10 (16.4)
Unknown reasons	6 (9.8)
Fetal distress	6 (9.8)
Placental detachment	5 (8.2)
Loss of end-diastolic flow in the umbilical artery	2 (3.3)
Uterine rupture	1 (1.6)
Hemorrhagic placenta previa totalis	1 (1.6)
Cord entanglement	1 (1.6)

 In Spearman’s correlation test, transition time to FEF was found to be positively associated with lactate level on admission (*r, P*: 0.295, 0.044), BUN (*r, P*: 0.285, 0.026) and creatinine levels (*r, P*: 0.345, 0.007) at the 24th hour of life. No correlations were found between transition time to FEF and any of the other laboratory results. Higher lactate, BUN, and creatinine values ​​were found to be probable risk factors leading to a longer transition time to FEF. Associations between laboratory findings with transition time to FEF are shown in Table 3.

**Table 3:** Associations between Laboratory Findings with Transition Time to Full Enteral Feeding in Neonates with Hypoxic-ischemic Encephalopathy (Spearman’s Correlation Test)

	**Transition Time to Full Enteral Feeding, Days**	
	***r***	***P***
pH, mm Hg	-0.237	0.066
Base excess, mmol/L	-0.183	0.158
Lactate, mmol/L	0.295	0.044*
Bicarbonate, mmol/L	-0.235	0.071
Carbon dioxide, mm Hg	0.128	0.326
Uric acid, mg/dL	0.007	0.96
Creatinine kinase, U/L	0.023	0.866
Creatinine kinase- muscle brain, ng/mL	-0.065	0.615
LDH, U/L	0.083	0.53
Troponin I, ng/mL	0.151	0.327
AST, U/L	0.160	0.219
ALT, U/L	0.128	0.325
BUN, mg/dL	0.285	0.026*
Creatinine, mg/dL	0.345	0.007*
PT, s	0.191	0.189
INR	0.205	0.158
aPTT, s	0.195	0.180
Hemoglobin, g/dL	-0.196	0.131
Leukocyte, /mm^3^	-0.047	0.716
Platelet, /mm^3^	-0.151	0.247

ALT, Alanine aminotransferase; AST, Aspartate aminotransferase; BUN, Blood urea nitrogen; PT, Prothrombin time; INR, international normalized ratio; aPTT, activated partial thromboplastin time; LDH, Lactate dehydrogenase. *P < 0.05.

 In Spearman’s correlation test, transition time to FEF was found to be negatively correlated with gestational week (*r, P*: -0.280, 0.029) and birth weight (*r, P*: -0.315, 0.013); and positively correlated with invasive mechanical ventilation days (*r, P*: 0.565, 0.0001), non-invasive mechanical ventilation days (*r, P*: 0.261, 0.042), antibiotic days (*r, P*: 0.556, 0.0001), inotropic agent days (*r, P*: 0.524, 0.0001) and hospitalization days (*r, P*: 0.654, 0.0001). Smaller GA, lower birth weight and worse clinical course (needing longer duration of mechanical ventilation, ​​antibiotic and inotrop use) were found to be probable risk factors leading to a longer transition time to FEF. Duration of hospital stay was found to be affected by the time to FEF. Associations between clinical findings with transition time to FEF are shown in Table 4.

**Table 4:** Associations between Clinical Findings with Transition Time to Full Enteral Feeding Neonates with Hypoxic-ischemic Encephalopathy (Spearman’s Correlation Test)

	**Transition Time to Full Enteral Feeding, Days**	
	***r***	***P***
Mother age	0.201	0.12
APGAR, first min	-0.303	0.097
APGAR, fifth min	-0.345	0.057
Gestational w	-0.280	0.029*
Birth weight, g	-0.315	0.013*
Invasive mechanical ventilation days	0.565	0.0001*
Non-invasive mechanical ventilation days	0.261	0.042*
Antibiotic days	0.556	0.0001*
Inotrope days	0.524	0.0001*
Hospitalization days	0.654	0.0001*

*P < 0.05.

## Discussion

 In our study, we confirmed that the transition time to FEF in neonates with moderate and severe HIE, who were treated with TH, was associated with both some laboratory results and the clinical course of the patients. At first, the GA and birth weight were inversely related to transition time to FEF, so when starting to feed those neonates, the basic most important thing is to take the GA and birth weight into consideration.

 When we analyzed the associations between transition time to FEF and laboratory results, we demonstrated that the transition time to FEF increased as lactate, creatinine and BUN levels increased. The lactate levels increase and the renal functions are impaired, if the oxygen level is not enough to meet the metabolic demands of the tissues and kidneys.^13^ Serum creatinine was found to correlate with HIE severity, because serum creatinine was found to be significantly elevated with the increase in HIE severity according to Sarnat and Sarnat staging, and higher serum lactate level following TH was found to be associated with poor neurodevelopmental outcome.^14,15^ It might be speculated that mesenteric blood flow was also impaired in those patients with higher lactate levels and impaired renal functions, so they needed longer to tolerate enteral feeding.

 We found that the duration of both invasive and non-invasive mechanical ventilation, antibiotic use, inotropic treatment and hospitalization were associated with transition time to FEF. It could be suggested that the more the neonate is stable, the shorter time it takes to transit to FEF. In a retrospective, matched case-control, exploratory pilot study, Chang et al^9^ found that feeding during TH was related to clinically significant reductions in the duration of the time to full oral feeds and in the length of hospital stay. In addition, they showed that no evidence indicating a rise in systemic inflammation was observed in the group that received feeds in spite of the similar extent of brain MRI injury in the MEN and unfed groups during TH. The scarce existing evidence suggests that careful introduction of enteral feeds during TH is safe, may advantageously modulate inflammatory responses and may be related to a shorter time to FEF and earlier discharge.^7^ Sakhuja et al^16^ reported that intestinal blood flow did not alter during the period of TH, but increased significantly after rewarming in neonates with HIE. The rewarming time was temporally related to a major increase in intestinal flow mainly due to increased heart rate and left ventricular output, without any increase in the left ventricular ejection fraction. They suggested that stable gastrointestinal hemodynamics during TH may show a favorable effect of cooling against reperfusion injury of the gastrointestinal tract as well as preventing reperfusion injury of the brain.^16^ It is not clear how to provide nutrition to infants with HIE, but it is essential to consider that enteral feeding could be an advantageous adjunct to TH, while parenteral nutrition may raise the risk of infection, though it is a reliable vehicle for nutrient delivery.^7,16,17^

 Minimal enteral nutrition during TH is the preferred feeding strategy in our clinic. As far as we know, our study is the first study that assessed the factors associated with the transition time to FEF in a group of neonates with HIE undergoing TH. The risk of NEC is known to be increased in babies with HIE, and mild to moderate TH was shown to protect against intestinal ischemia-reperfusion injury.^4,13,18^ In a pilot study, it was shown that cooling was reasonable and safe for treatment of preterm infants with NEC complicated by multiple organ dysfunction syndrome.^19^ MEN during TH shortens the duration of the time to FEF and hospitalization period, and is not related to an increase in mortality or morbidity, though the optimum nutritional strategy for infants with HIE during TH is unclear.^6,8,9^

 Our study has some limitations. The first of the main limitations of our study was the relatively small sample size. Secondly, the retrospective cross sectional design did not allow us to compare the neonates in a case-control fashion. Finally, we could not obtain the data about type of feeds (expressed maternal breast milk or artificial formula) that might have an effect on feeding tolerance.^20^ The major strength of our study was the fact that a standardized feeding protocol was utilized which resulted in similar feed volumes and daily increments.

 In conclusion, the findings of our study show that clinicians should be more careful when starting to feed babies undergoing TH with higher lactate levels and impaired renal functions as these findings might be associated with feeding intolerance and severity of HIE. Clinically stable neonates with less severe HIE could tolerate enteral feeding while on TH, and clinicians should be encouraged to feed those babies as soon as possible. Further randomized prospective trials are warranted to clarify the optimum enteral feeding strategy for neonates with HIE undergoing TH.
